# Transgenic Analyses in Drosophila Reveal That mCORL1 Is Functionally Distinct from mCORL2 and dCORL

**DOI:** 10.1534/g3.119.400647

**Published:** 2019-09-17

**Authors:** Michael J. Stinchfield, Keiji Miyazawa, Stuart J. Newfeld

**Affiliations:** * School of Life Sciences, Arizona State University, Tempe, AZ 85287-4501 and; † Department of Biochemistry, University of Yamanashi, Chuo, Yamanashi, 409-3898 Japan

**Keywords:** Fussel/SKOR/CORL, gene duplication, mushroom body neurons, neofunctionalization, Sno homology domain

## Abstract

Uncovering how new members of multigene families acquire new functions is an important topic in evolutionary and developmental genetics. CORL proteins (SKOR in mice, Fussel in humans and fussel in Flybase) are a family of CNS specific proteins related to mammalian Sno/Ski oncogenes. Drosophila CORL (dCORL) participates in TGF-β and insulin signaling during development and in adult homeostasis but roles for the two mouse CORL proteins (mCORL) are essentially unknown. A series of studies were conducted to test the hypothesis based on previous results that mCORL1 is more similar to dCORL than mCORL2. Neither an updated alignment nor ectopic expression in adult wings were able to distinguish mCORL1 or mCORL2 from dCORL. Transgene experiments employing a dCORL endogenous function in mushroom body neurons showed that mCORL1 is distinct from mCORL2 and dCORL. mCORL1 and mCORL2 are also distinct in biochemical assays of Smad-binding and BMP signaling. Taken together, the data suggests testable new hypotheses for mCORL2 function in mammalian TGF-β and insulin signaling based on known roles for dCORL. Overall, the study reiterates the value of transgenic methods in Drosophila to provide new information on multigene family evolution and the function of family members in other species.

The dominant model for the growth of multigene families and the acquisition of novel functions by new genes is based on gene duplication with one copy of the pair maintaining the original function while the other copy accumulates advantageous mutations leading to a novel function. We recently explored the consequences of this ‘duplication then neofunctionalization’ model for new genes in mammals and flies. For each, we employed the conserved Dpp/BMP signaling pathway (subfamily of the TGF-β family) that regulates embryonic dorsal-ventral axis formation in flies and vertebrates as our assay. Neofunctionalization for two new genes contrasted with the conservation of a third new gene.

First, in mammals the new gene TRIM33 is not present in flies. It encodes a RING class ubiquitin ligase that monoubiquitylates the BMP signal transducer Smad4 during dorsal-ventral patterning (Wisotzkey *et al.* 2014). Second, in insects the new gene *lolal* is not present in mammals. It encodes a BTB domain protein that functions in a chromatin-remodeling complex maintaining Dpp transcription during dorsal-ventral axis formation (Quijano *et al.* 2016). Third, the Smad4 deubiquitinase USP9X in mammals and its fly homolog *fat facets* are highly conserved. These genes both remove a monoubiquitin from the same lysine in Smad4/Medea during dorsal-ventral pattern formation in their respective species (Stinchfield *et al.* 2012).

For the CORL family, we examine the influence of sequence conservation on functional conservation across flies and mammals. We shift our experimental focus, but remain within TGF-β signaling, by analyzing the Activin pathway (sister to the Dpp/BMP pathway) in the larval brain. In Drosophila mushroom body neurons dSmad2 signaling upstream of EcR-B1 transcriptional activation is facilitated by the Smad binding dCORL protein (Takaesu *et al.* 2012). dCORL belongs to a family of Smad interacting proteins that include the mammalian SnoN and c-Ski protooncogenes that share a Smad binding region called the Sno homology domain (Takaesu *et al.* 2006). dCORL has two mouse relatives in this family.

mCORL1 (mSKOR1) was identified as a Sno/Ski family member that functions as a transcriptional co-repressor in cell culture. In embryos mCORL1 is expressed only in dorsal interneurons of the cerebellum (Mizuhara *et al.* 2005). In embryos mCORL2 (mSKOR2) is expressed only in Purkinje neurons of the cerebellum (Minaki *et al.* 2008). Loss of function studies of mSKOR2 revealed a requirement for Purkinje cell differentiation (Miyata *et al.* 2010; Wang *et al.* 2011). mSKOR2 knockouts demonstrated that this is accomplished by inhibiting interneuron fate (Nakatani *et al.* 2014). No knockout studies of mSKOR1 have been reported. mSKOR1 is primarily, though not exclusively, present in the cerebellum of adults. mSKOR2 is restricted to the cerebellum in adults though the cell types are unknown (Yue *et al.* 2014). The sequence conservation and common CNS specificity of CORL proteins suggests that transgenic analysis of mCORL1/2 in flies will suggest new hypotheses for their developmental roles.

Transgene studies of mCORL1, mCORL2 and dCORL will also allow us to address the related evolutionary question, has one of the recently duplicated mCORL proteins acquired a distinct function? Functional divergence can be detected in two ways. One way is by rescuing a mutant phenotype in one species with a family member from another. For example, human BMP2 and BMP4 are 96% similar to each other in the ligand domain (102 residues) and both rescue Dpp mutants during dorsal-ventral patterning. Underlying the rescue, BMP2 is 82% and BMP4 is 81% similar to Dpp in the ligand region (Padgett *et al.* 1993).

A second means of identifying neofunctionalization is by comparing phenotypes generated in parallel transgene assays. For example, a single amino acid change created a new function in a human Smad tumor allele (hSmad4^R100T^; Takaesu *et al.* 2005). Another study revealed that hSmad2 and hSmad3 generated distinct phenotypes. hSmad2 phenotypes were consistent with dSmad2 while hSmad3 phenotypes were distinct (Marquez *et al.* 2001). Sequence comparison of hSmad2 and hSmad3 revealed eight amino acid substitutions in a conserved DNA-binding domain that were likely responsible for the distinction.

Here we demonstrate that mCORL1 has a distinct function from mCORL2 and dCORL with the latter two presumably sharing the ancestral function. Taken together, the data suggests testable new hypotheses for mCORL2 function in mammalian TGF-β and insulin signaling based on known roles for dCORL. Overall, the study reiterates the value of transgenic methods in Drosophila to provide new information on multigene family evolution and the function of family members in other species.

## Materials and Methods

### Bioinformatics

Similarity calculations were derived from alignments of the primary ORF for dCORL, (JX126878), mCORL1 (AK049035) and mCORL2 (NM_001109743) generated with Clustal Omega at www.ebi.ac.uk/Tools/msa/clustalo/ as described (Wisotzkey *et al.* 2014). The Sno homology domain alignment illustration was generated in Boxshade at ch.embnet.org/software/BOX_form.html as described (Kahlem and Newfeld 2009).

### Drosophila Genetics

Wing experiment stocks were: MS1096.Gal4 (Marquez *et al.* 2001), UASt.dCORL (insertions on II and III; Takaesu *et al.* 2012), UASt.mCORL1 (insertions on X, II and III) and UASt.mCORL2 (insertions on X, II and III). Crosses for wing expression were kept at 25°.

Flip out clone stocks were: yw hs.FLP; AY.Gal4 UASt.GFP; *Df(4)dCORL* / ci^*D*^ (Struhl and Basler 1993; Takaesu *et al.* 2012) alone as a control or crossed to: 1) UASt.dCORL on III; *Df(4)dCORL*/*ci^D^*, 2) UASt.mCORL1 on III; *Df(4)dCORL*/*ci^D^* or 3) UASt.mCORL2 on III; *Df(4)dCORL*/*ci^D^* for rescue and overexpression experiments. Fly crosses for rescue and overexpression experiments were maintained at 25° prior to heat shock. Larvae were heat shocked 40-64 hr after egg lay for 1 hr @ 37°. After one hour of recuperation at 18° they were returned to 25°. Wandering 3^rd^ instar larvae were picked 6 days after egg lay and checked for GFP before dissection (Takaesu *et al.* 2012). After staining, EcR-B1 expression in the lobe without a clone in the MB was employed to determine *Df(4)dCORL* status (33% homozygous mutant or 66% heterozygous with ci^*D*^ that were effectively wild type; ci^*D*^ homozygotes die as embryos). This allowed the interpretation of the lobe with a clone in the MB as either mutant rescue or wild type repression of EcR-B1. At least seven brains were examined per genotype (8 genotypes total). The number of brains with clones in the MB for each genotype is reported in the figure legend for each experiment.

### Immunofluorescence

Post-heat shock larvae that have stopped wandering but not yet begun pupariation (prior to anterior spiracle eversion - equivalent to 122 hr at 25° for *yw*) were picked, sorted and their brains dissected as in Tran *et al.* (2018a,b). Brains were fixed in 4% formaldehyde, rinsed, and stored in methanol at -20°. Primary antibodies were mouse α-EcR-B1 (DSHB AD4.4), guinea pig α-Tailless (Kosman *et al.* 1998; gift of Dr. John Reinitz), and rabbit α-GFP (Abcam). Secondary antibodies were goat α-mouse, α-rabbit, α-guinea pig Alexa Fluor 488, 546 and 633 (Molecular Probes). Brains were mounted in 90% Glycerol/PBS and imaged on a Leica SP5. Pixel intensity for EcR-B1 expression in clones was assayed as described (Quijano *et al.* 2016).

### Molecular Biology

cDNAs for mCORL1 (Isoform2 NP_001157227.1, pMX-FLAG NII-mCORL1; Mizuhara *et al.* 2005) and mCORL2 (NP_001103213.1, pMX-FLAG NII-mCORL2, Minaki *et al.* 2008) were a kind gift of Dr. Yuichi Ono (Kan Research Institute, Kobe, Japan). mCORL1 Isoform2 differs from the longest version (Isoform1) via alternative exons eight amino acids downstream of the initiator methionine. The next 34 amino acids in Isoform1 are replaced by six amino acids in Isoform2. The remainder of the coding region is identical. Isoform3 is likely an artifact as it is identical to Isoform2 but missing a single glutamic acid near the carboxy-terminus. Isoform4 may also be an artifact as it initiates at an internal methionine 39 amino acids downstream of the methionine shared by the other 3 isoforms. The amino-terminal variable region is not conserved in dCORL nor mCORL2 and the Sno homology domain is intact in all isoforms suggesting the small differences do not impact mCORL1 function. mCORL2 has only a single isoform.

Each open reading frame was amplified by PCR and a Kozak consensus sequence for eukaryotic translation initiation added upstream of the initiator methionine. mCORL1 was cloned into the EcoRI - AccI sites and mCORL2 into the EcoRI - SfiI sites of pUASt (Brand and Perrimon 1993). Fly transformation and transformant mapping were via standard methods.

Immunoprecipitation and immunoblotting were as described (Motizuki *et al.* 2013). 293T cells were transfected using X-treamGENE9 and lysates subject to immunoprecipitation using anti-FLAG-M2 (Sigma, St. Louis, MO). For the analysis of the Flag-mCORL2 and Myc-hSmad3 interaction, with and without the human constitutively active TGF-β receptor Alk-5-TD-HA, IP was followed by immunoblotting with α-Myc 9E10 (Santa Cruz Biotech, Dallas, TX). Filters were directly blotted with α-Myc, α-FLAG, α-HA or α-tubulin as loading controls. An analysis of Flag-cSki (human) was conducted in parallel as a positive control.

Transcriptional activation was measured as described (Motizuki *et al.* 2013) in the presence of human constitutively active TGF-β receptor Alk5-TD-HA or human constitutively active BMP receptor ALK3-QD-HA with 12xCAGA-Luc and BRE-Luc luciferase reporter constructs containing TGF-β-responsive or BMP-responsive elements, respectively (Korchynskyi and ten Dijke 2002; Dennler *et al.* 1998). HepG2 cells were seeded in 24-well plates and transiently transfected with mCORL1, mCORL2 or c-Ski (mouse as a positive control) and paired receptor-reporter constructs. Cell lysates were prepared and luciferase activities were measured with the Dual-Luciferase reporter system (Promega, Madison, WI) using a luminometer (MicroLumat Plus, Berthold, Bad Wildbad, Germany). Values were normalized to co-transfected Renilla luciferase under control of the thymidine kinase promoter.

### Data Availability

Strains and clones are available upon request. The authors affirm that all data necessary for confirming the conclusions of the article are present within the main article, figures and table plus the supplemental figures and table. Supplemental material available at FigShare: https://doi.org/10.25387/g3.9862838.

## Results

### mCORL1 and mCORL2 are equidistant from dCORL in the Sno homology domain

**Figure 1:**
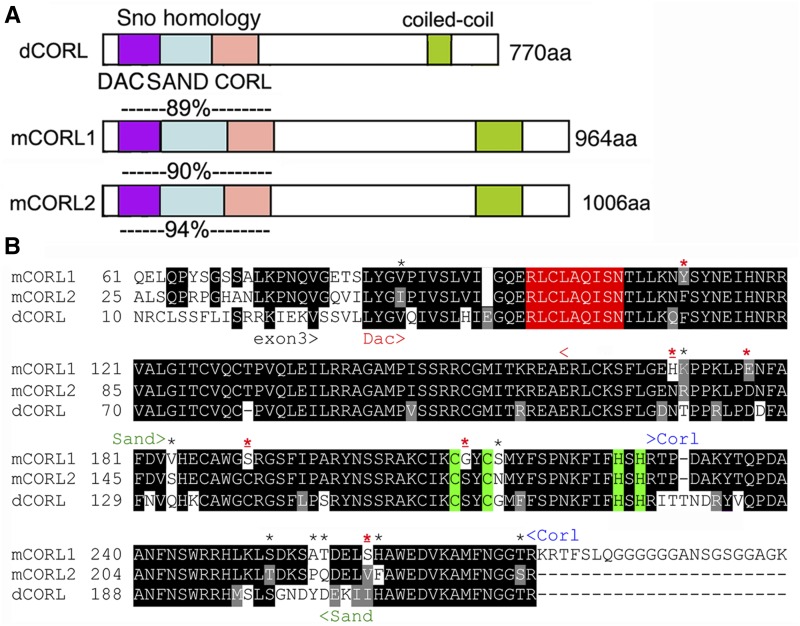
mCORL1 and mCORL2 are equidistant from dCORL in the Sno homology domain. A) Schematic of CORL proteins from fly and mouse with the locations of five named domains shown. Amino acid similarity in the Sno homology domain (Smad binding; 195 residues) between dCORL and mCORL1 is shown above mCORL1, between dCORL and mCORL2 above mCORL2 and between mCORL1 and mCORL2 below mCORL2. Similarity is the sum of identical residues and conservative substitutions where both amino acids share biochemical properties: D/E, K/R/H, N/Q, S/T, I/L/V, F/W/Y, A/G (Smith and Smith 1990). B) Sno homology domain alignment. An amino acid is shaded if the residue is identical (black) or similar (gray) in two or three proteins. The APC recognition site is red and Cys2-His2 zinc finger in green. The Dac, Sand and CORL domains are shown with arrowheads. Fourteen amino acids different between mCORL1 and mCORL2 are indicated by *, a red * indicates 6 positions where dCORL and mCORL2 both differ from mCORL1 and red * indicates 4 positions where mCORL1 has a biochemically distinct amino acid from both dCORL and mCORL2.

The premise that similar sequences lead to similar functions is the reason investigators use BLAST to search for proteins similar to their favorite protein with the hope that functional data exists for a match, pointing to a specific hypothesis they can test in their system. A tree of the CORL/Sno/Dac family that share the Smad-binding Sno homology domain was valuable in suggesting we look in mushroom body neurons for dCORL function as this was the only established location for Activin subfamily signaling at the time (Takaesu *et al.* 2102). A schematic of the structure of CORL proteins and an assessment of the similarity of dCORL, mCORL1 and mCORL2 in the Smad-binding Sno homology domain based on a new alignment is in Figure 1A.

The amino acid similarity (defined as identical plus conservative substitutions), between the two mCORL proteins and dCORL in the Sno homology domain is greater than initially reported due to updated methods underlying the new alignment. In this region mCORL1 and mCORL2 (94% similar with 14 differences over 195 residues) are almost as similar as human BMP2 and BMP4 (96%) in the ligand region. The conservation of these two with dCORL, 89% for mCORL1 (27 differences) and 90% for mCORL2 (24 differences) is very high. This is greater than the conservation of Dpp with BMP2 (82%) or BMP4 (81%), proteins that can reciprocally substitute for each other across species (Padgett *et al.* 1993, Sampath *et al.* 1993).

Taking a closer look at the alignment of mCORL1, mCORL2 and dCORL, we note there are fourteen differences between mCORL1 and mCORL2 (Figure 1B). Of these there are six locations where dCORL and mCORL2 are similar and both differ from mCORL1. While the alignment shows that mCORL1 is slightly more distant from dCORL and mCORL2, it is not a significant difference. Thus, we began the transgenic analysis with the hypothesis based on a previous report of shared function for mCORL1 (binds Smad3 in the Activin pathway) and dCORL (upstream of EcR-B1 transcription in the Activin pathway) as most similar.

### mCORL1 and mCORL2 are not clearly distinguished from dCORL in ectopic wing assays

To test the “mCORL1/dCORL more similar hypothesis” we generated UASt transgenes expressing mCORL1 and mCORL2 and created fly stocks with insertions on multiple chromosomes. Similar dCORL transgene stocks with insertions on several chromosomes already exist (Takaesu *et al.* 2012). For the initial side-by-side test we chose a familiar location - the wing (*e.g.*, Marquez *et al.* 2001, Takaesu *et al.* 2005, Quijano *et al.* 2011). Employing wings is further supported by the fact that *dCORL* has no endogenous wing expression leading to all three CORL proteins being ectopic. In prior wing assays we found that dCORL ectopic expression can interfere with Dpp signaling visible as vein truncations. Since loss of function studies revealed that the true role of dCORL is to facilitate Activin signaling, we hypothesized that Dpp antagonism resulted from non-specific interference with Mad (Takaesu *et al.* 2012).

**Figure 2:**
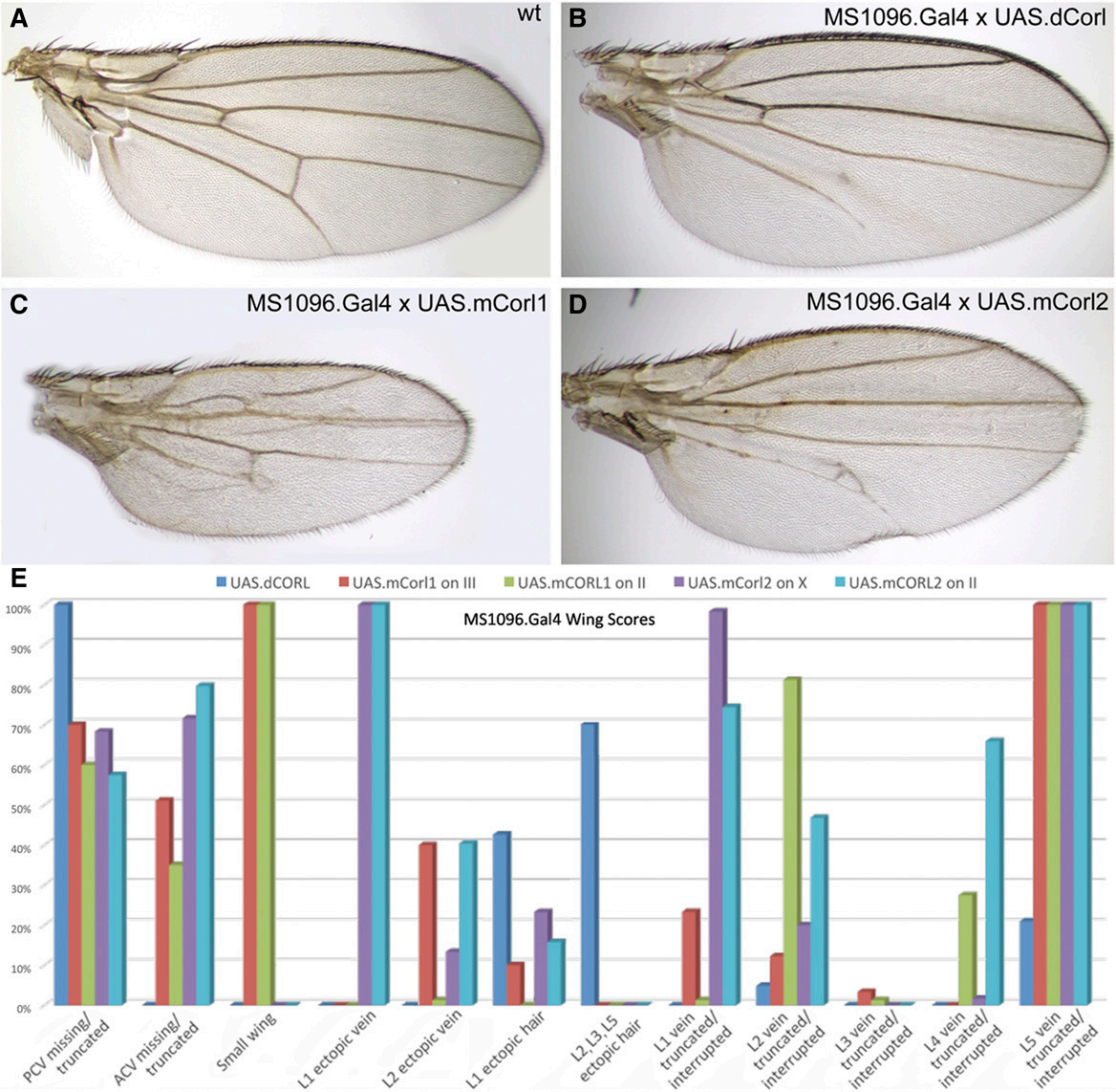
mCORL1 and mCORL2 are not clearly distinguished from dCORL in wing assays. A) Wild type adult female wing. B) UASt.dCORL wing is normal size with 4 ectopic margin bristles on longitudinal vein1, missing posterior crossvein and truncated longitudinal vein5. C) UASt.mCORL1 wing is on average 40% smaller than wild type, has an interrupted longitudinal vein2, missing anterior crossvein and truncated longitudinal vein5. D) UASt.mCORL2 wing with a bifurcated longitudinal vein4, missing anterior crossvein and delta at the posterior crossvein. E) Graph of all phenotypes scored for dCORL and two insertions each of mCORL1 and mCORL2. Of 12 phenotypes, four are in common, four are found only in mCORL1 and mCORL2 and there is one each unique to mCORL1 and mCORL2. Numerical data in Table S1.

Our studies employed the wing blade driver MS1096.Gal4 and we scored only female wings (*e.g.*, Quijano *et al.* 2010). All three CORL proteins produced wings with vein truncations at a significant frequency and phenotypes generated by different insertions for each transgene were consistent (Figure 2). The geography of vein defects was overall quite similar for all CORL proteins. For example, defects in the anterior and posterior crossveins, and truncations in longitudinal vein5 were present in all genotypes (Figure 2E). Each of the mCORL transgenes distinguished itself from the others in a single phenotype. For mCORL1 it was small wing and for mCORL2 it was ectopic vein tissue protruding from longitudinal vein1. Pixel occupancy in photos from wings of mCORL1 (n = 7) were on average 40% smaller than dCORL (n = 7) even though RT-PCR indicated roughly equal expression (Table S1). No size difference between the dCORL and mCORL2 (n = 10) was detected.

Interestingly, a phenotype that distinguishes dCORL from both mCORL transgenes is the strength of interference with Mad’s Dpp-independent role in sensory organ precursor self-renewal (Quijano *et al.* 2011). Ectopic margin bristles on longitudinal veins2, 3 and 5 are seen only with dCORL. Notwithstanding this last point, the original hypothesis that mCORL1 will function more similarly to dCORL than mCORL2 is not supported by ectopic wing studies.

### Rescue of EcR-B1 in the MB of dCORL mutants by mCORL2/dCORL not mCORL1

Previously, to show that the loss of EcR-B1 mushroom body (MB) expression in *Df(4)dCORL* mutant brains was due to the loss of *dCORL*, we rescued EcR-B1 expression with heat shock driven flip-out clones of UASt.dCORL (Takaesu *et al.* 2012). This rescue assay is arguably the most rigorous test possible of the hypothesis that mCORL1 is more similar in function to dCORL than mCORL2. A rescue assay of an endogenous function for dCORL is analogous to the BMP2/4 transgene rescue of the dorsal-ventral defect in Dpp mutants (Padgett *et al.* 1993). We examined flip-out clones in third instar brains of *Df(4)dCORL* mutants expressing UASt.GFP or UASt.GFP with either UASt.dCORL or UASt.mCORL1 or UASt.mCORL2 (the latter were all inserted on chromosome III and employed in the wing assay).

**Figure 3:**
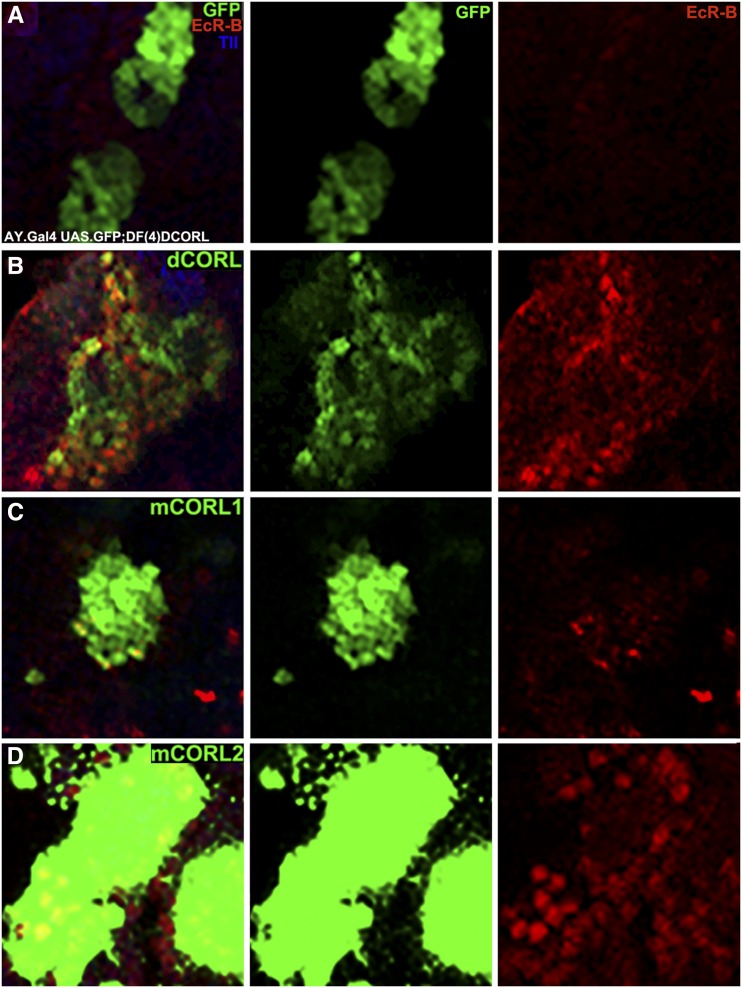
Rescue of EcR-B1 in the MB of *dCORL* mutants by mCORL2/dCORL not mCORL1. High magnification dorsal view of mushroom body neurons in *Df(4)dCORL* brains with anterior up. An image of a single slice is shown from left to right as three color (includes Tailless in blue), green alone (GFP), and red alone (EcR-B1). Genotype determined by reference to the sister brain lobe and clone location by reference to Tailless in MB neuroblasts not affected in *Df(4)dCORL*. A) A clone expressing AY.Gal4 UASt.GFP on II alone. EcR-B1 is absent (n = 6). B) A clone expressing AY.Gal4 UASt.GFP on II and UASt.dCORL on III. EcR-B1 is rescued (n = 8). C) A clone expressing AY.Gal4 UASt.GFP on II and UASt.mCORL1 on III. EcR-B1 expression is faint (n = 12). D) A clone expressing AY.Gal4 UASt.GFP on II and UASt.mCORL2 on III. EcR-B1 is rescued, a phenocopy of dCORL (n = 8). Numerical data in Table 1.

The data are shown at high magnification in Figure 3. For perspective on clone size, location and numbers each whole brain lobe is shown in Fig. S1. Clones marked with GFP alone in *Df(4)dCORL* brains are easily visible in the presumptive EcR-B1 domain of the MB body. This location is characterized by proximity to Tailless expressing MB neuroblasts that are unaffected in *Df(4)dCORL* brains (Takaesu *et al.* 2012). As a negative control, GFP alone does not rescue EcR-B1 expression in mutants with clones (Figure 3A; quantification in Table 1). As a positive control, co-expressing dCORL, with GFP to mark the clone, in *Df(4)dCORL* brains fully rescues EcR-B1 (Figure 3B). Quantification of pixel intensity shows that rescued EcR-B1 expression is not substantially different from wild type (Table 1). We identify a brain as a rescued mutant and not a heterozygous brain with wild type EcR-B1 by the absence of EcR-B1 in the sister brain lobe that has no clones in the MB. In the experiment, GFP alone is the negative control and dCORL the positive control for mCORL1/2 influence on EcR-B1.

**Table 1:** Quantification of EcR-B1 expression in rescue and overexpression clones

Rescuing transgene (**Fig. 3**)	Mean pixel intensity	EcR-B1 status	Overexpressing transgene (**Fig. 4**)	Mean pixel intensity	EcR-B1 status
GFP	4.34	off	GFP	42.027	on
dCORL	35.124	rescue	dCORL	16.459	repress
mCORL1	16.255	half-rescue	mCORL1	64.125	on
mCORL2	29.467	rescue	mCORL2	28.118	half-repress

In this assay, MB clones of mCORL1 did not fully rescue EcR-B1 expression (Figure 3C). Faint EcR-B1 expression is visible that is roughly half of that seen in dCORL clones (Table 1). Clones of mCORL2 were able to fully rescue EcR-B1 matching the ability of dCORL (Figure 3D; quantification in Table 1). These results suggest that mCORL1 is distinct from mCORL2/dCORL, contrary to our initial hypothesis and in favor of an alternative hypothesis, that mCORL2 is more similar to dCORL.

### Repression of EcR-B1 in the MB of wild type by mCORL2/dCORL not mCORL1

Pursuing another line of experimentation employed in our prior analysis of dCORL, we examined flip-out clones expressing dCORL, mCORL1 and mCORL2 in the MB of wild type siblings of *dCORL* mutants (these are heterozygous over ci^*D*^ and this is reasonable since there is no evidence from stage of lethality studies for haploinsufficiency of *Df(4)dCORL*; Takaesu *et al.* 2012). Expression of dCORL from flip-out clones in a wild type brain results in overexpression for an endogenous function. Previously we showed dCORL overexpression results in the loss of EcR-B1 expression (Takaesu *et al.* 2012). We believe this phenotype is due to excess dCORL interfering with Activin signaling, analogous to dCORL antagonism of Dpp signaling when ectopically expressed in wing disks.

**Figure 4:**
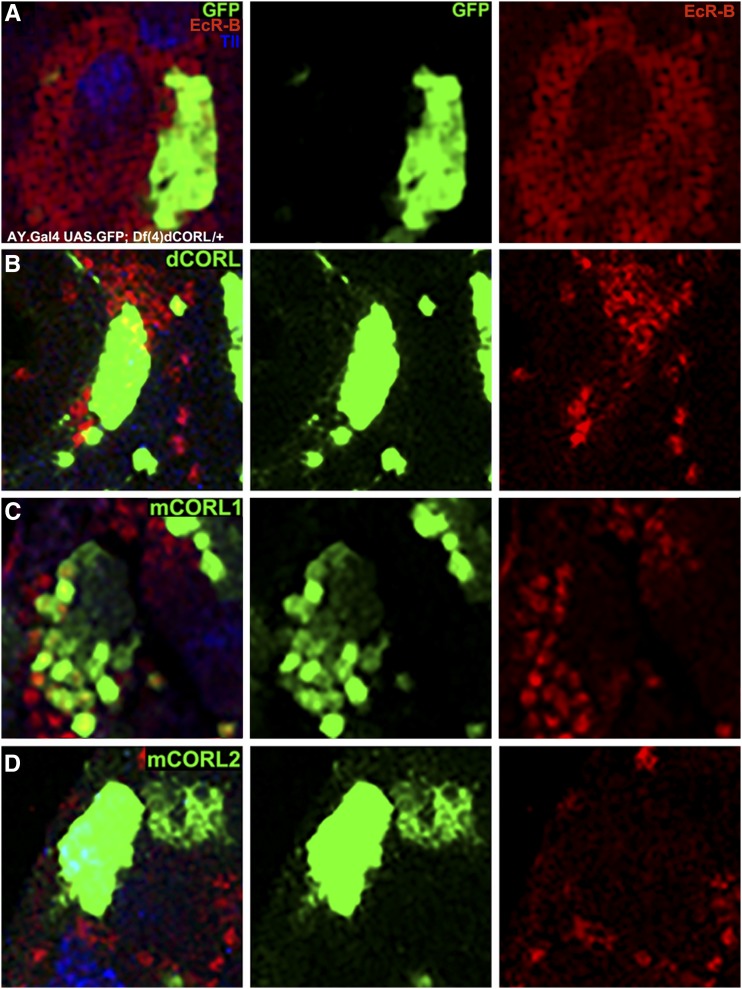
Repression of EcR-B1 in the MB of wild type by mCORL2/dCORL not mCORL1. High magnification dorsal view with anterior up of mushroom body neurons in the brains of wild type siblings (*Df(4)dCORL*/+) of the mutants shown in Fig. 3. An image of a single slice is shown from left to right with genotype and clone location determined as in Fig. 3. A) A clone expressing AY.Gal4 UASt.GFP on II alone. EcR-B1 is unaffected (n = 6). B) A clone expressing AY.Gal4 UASt.GFP on II and UASt.dCORL on III. EcR-B1 is reduced in the clone (n = 4). C) A clone expressing AY.Gal4 UASt.GFP on II and UASt.mCORL1 on III. Where EcR-B1 overlaps the clone it is largely unaffected (n = 8). D) A clone expressing AY.Gal4 UASt.GFP on II and UASt.mCORL2 on III. EcR-B1 is reduced in the clone, a phenocopy of dCORL (n = 15). Numerical data in Table 1.

The data are shown at high magnification in Figure 4. For perspective, each whole brain lobe is shown in Fig. S2. Clones marked with GFP alone in wild type brains are easily visible in the presumptive EcR-B1 domain of the MB body. As a negative control, GFP clones do not repress EcR-B1 expression in wild type (Figure 4A; quantification Table 1). As a positive control, co-expressing dCORL, with GFP to mark the clone, in wild type brains represses EcR-B1 to roughly a third of wild type (Figure 4B; Table 1). We identify brains as repressed wild type and not a mutant brain with missing EcR-B1 function by the presence of EcR-B1 expression in the sister brain lobe that has no clones in the MB region. GFP alone is the negative control and dCORL the positive control for mCORL1/2.

In this assay, MB clones of mCORL1 did not repress EcR-B1 expression (Figure 4C; Table 1). Clones of mCORL2 in the MB were able to repress EcR-B1 to roughly two-thirds of wild type, nearly matching the ability of dCORL (Figure 4D; Table 1). The results from overexpression clones are consistent with the rescue data for EcR-B1 in *dCORL* mutants. Genetic evidence from both assays of an endogenous function of dCORL (regulation of EcR-B1 expression) support the alternative hypothesis that mCORL1 has a divergent function with mCORL2 and dCORL more similar.

### mCORL2 biochemical functions are distinct from mCORL1

Previously, we showed that mCORL1 bound strongly to mSmad3 (dSmad2 homolog) consistent with loss of EcR-B1 expression in *dCORL* mutants (Takaesu *et al.* 2012). Thus we took this approach again. We analyzed mCORL2 under the same conditions in binding assays with mSmad3 and luciferase assays with TGF-β/Activin and BMP specific reporters.

**Figure 5:**
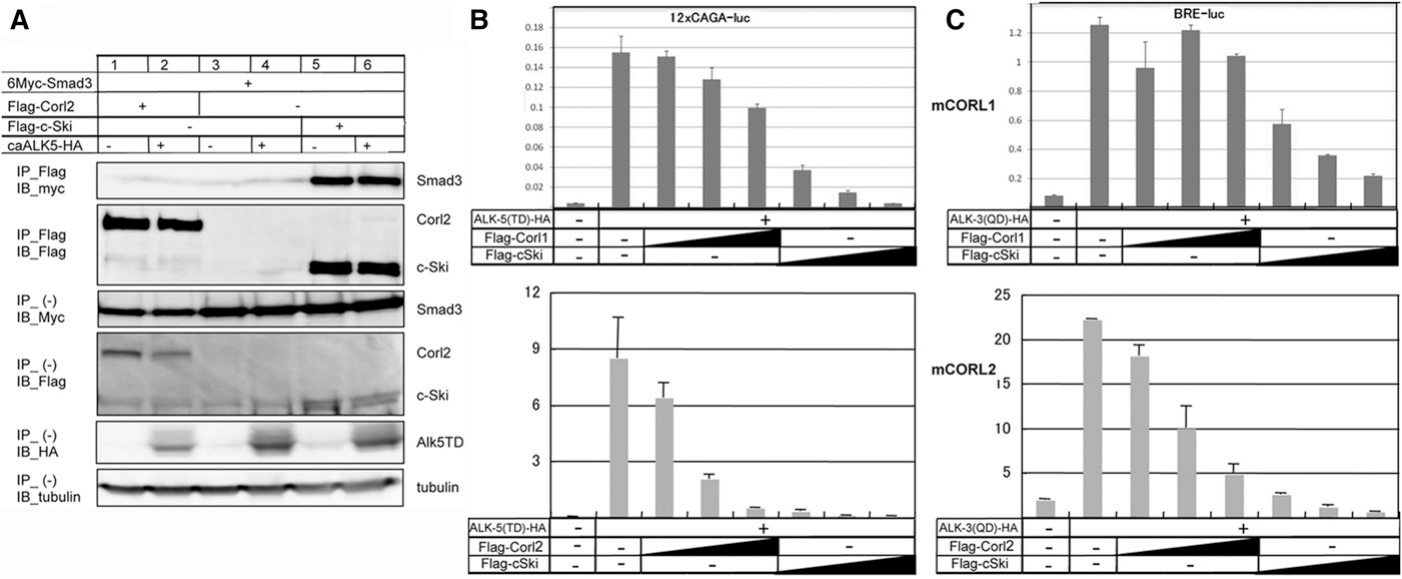
mCORL2 is biochemically distinct from mCORL1. A) Interaction of FLAG-tagged mCORL2 and c-Ski with 6Myc-tagged Smad3 was examined by IP followed by IB in 293T cells. HA-tagged constitutively active ALK-5 (caALK-5) was utilized to activate TGF-β signaling. The top panel shows the interaction of c-Ski with Smad3, but no interaction between mCORL2 and Smad3 (lane 2). The lower five panels are controls for protein expression. B) mCORL1 (top), mCORL2 (bottom) and c-Ski (top and bottom) reduce TGF-β signaling with increasing efficacy as shown by a 12xCAGA-Luc reporter stimulated with caALK-5 in HepG2 cells (in duplicate, error bars are shown). Activity was normalized against co-transfected Renilla luciferase and reported in arbitrary units. mCORL1 and mCORL2 are effective but not as effective as c-Ski at antagonizing TGF-β signaling. C) The same experiment for BMP signaling via a BRE-Luc reporter stimulated with caALK-3. mCORL1 (top) is distinct from mCORL2 (bottom) and c-Ski (top and bottom). As shown in the top, lanes 3-5 do not have a continuously downward trajectory indicating that mCORL1 cannot antagonize BMP signaling. mCORL2 and c-Ski can, with c-Ski a more effective antagonist than mCORL2 as shown by the relative heights of bottom lanes 3-5 (mCORL2) *vs.* 6-8 (c-Ski).

The binding data shows that mCORL2 does not bind Smad3 (Figure 5A) even in the presence of an activated TGF-β receptor. The region of CORL proteins that bind Smads is the Sno homology domain, based on conservation in this region with c-Ski and SnoN. This result points to the Sno homology domain as a source of distinction between mCORL1 and mCORL2.

Consistent with the binding assay, parallel studies of TGF-β and BMP signaling showed that mCORL1, mCORL2 and c-Ski can repress TGF-β signaling (Figure 5B) but only mCORL2 and c-Ski can repress BMP signaling in a dose-dependent manner (Figure 5C). We are aware of the inconsistency between mCORL1 luciferase data (fails to antagonize BMP) and the ectopic wing data (able to antagonize Dpp). We reconcile these by noting that the dosage of mCORL1 in the luciferase assays approximates physiological levels and is tightly controlled while in ectopic wing assays expression could be orders of magnitude above physiological levels. Thus, in the wing assays the overabundance of mCORL1 could lead to non-specific binding of Mad that does not occur in the luciferase assays. The biochemical data suggest that mCORL1 has a distinct function from mCORL2 as a result of amino acid changes in the Sno homology domain.

Taken together the sequence, genetic and biochemical data support the alternative hypothesis that dCORL and mCORL2 are the closest functionally with mCORL1 divergent. The data does not address which mCORL came first, as either of the two duplicates could diverge while the other maintained the ancestral function.

## Discussion

None of the data supported the initial hypothesis that mCORL1 is more similar to dCORL than mCORL2. Instead most experimental data were consistent with the alternative hypothesis that mCORL1 has a distinct function from mCORL2 and dCORL: 1) rescue of EcR-B1 in *dCORL* mutants, 2) repression of EcR-B1 when overexpressed in wild type, 3) mCORL2 failure to bind the mCORL1 partner mSmad3 and 4) mCORL1 inability to antagonize BMP signaling in luciferase assays. We recognize that the data are not black and white with similarities between mCORL1 and mCORL2 in the wing assays and modest rescue of EcR-B1 by mCORL1 in *dCORL* mutants. However, the preponderance of evidence indicates that mCORL2 and dCORL share the ancestral function and that mCORL1 has a distinct function.

From a larger perspective the data informs our understanding of multigene family evolution, specifically the accumulation of advantageous mutations. The six differences between the mCORL genes in the Sno homology domain are quantitatively similar to the eight differences between human Smad2 and Smad3 in the DNA-binding domain (Marquez *et al.* 2001). Both are consistent with the idea that a small number of amino acid differences in an otherwise highly conserved functional domain can confer distinct activities.

Regarding functions for mCORL2 based on its biochemical properties and the reported roles of dCORL (Takaesu *et al.* 2012; Tran *et al.* 2018b) several hypotheses are now explicit: mCORL2 may facilitate TGF-β/Activin signaling during development or cooperate with POU domain transcription factors in regulating the insulin pathway. SKOR2 knockout mice with Cre-Lox capabilities could provide a tractable system in which to test these hypotheses. Further, given the ability of mCORL2 to inhibit TGF-β and BMP signaling in luciferase assays, perhaps mCORL2 functions like a pathway switch (as shown for dSno; Takaesu *et al.* 2006).

In summary, our data that mCORL2 and dCORL share functions while mCORL1 has diverged reinforces the current working hypothesis that neofunctionalization of a new gene can be achieved with a handful of advantageous amino acid changes in a highly conserved functional domain. We also encourage our colleagues to experimentally test roles for mCORL2 in development and adult homeostasis that reflect functions for dCORL. Overall, the study reiterates the value of transgenic methods in Drosophila to provide new information on multigene family evolution and the function of family members in other species.
